# How Super Is Supertrack? Expediting Care of Fast-track Patients through a Pediatric Emergency Department

**DOI:** 10.1097/pq9.0000000000000770

**Published:** 2024-09-18

**Authors:** Daniel Lam, Cortney Braund, Sarah Schmidt, Bernadette Johnson, Sandra P. Spencer, Chisom Agbim

**Affiliations:** From the * Division of Pediatric Emergency Medicine, University of California San Francisco, Department of Emergency Medicine, San Francisco, Calif.; † Section of Emergency Medicine, Children’s Hospital of Colorado, Department of Pediatrics, University of Colorado School of Medicine, Aurora, Co.; ‡ Section of Emergency Medicine, Stanford University School of Medicine, Department of Pediatrics, Palo Alto, Calif.

## Abstract

**Background:**

Fast-track models decrease patient crowding in emergency departments (EDs) by redirecting low-acuity patients to an expedited care pathway. In 2016, this institution’s pediatric ED created a fast-track pathway for patients evaluated in a rapid assessment triage area who needed further management in the primary ED. This “Supertrack” designation was intended for patients requiring up to 1 hour of additional care, though means of ensuring these patients were discharged within their anticipated timeframe were lacking.

**Methods:**

We aimed to increase the percentage of Supertrack patients discharged within 1 hour of their ED bed assignment from 17% to 50%. Interventions included the creation of objective Supertrack criteria, departmental-wide progress reports, personalized reminders, intake huddles, and documentation prompts. We visualized data from Plan, Do, Study, Act (PDSA) cycles with statistical process control charts to determine special cause variation.

**Results:**

The percentage of Supertrack patients discharged within their anticipated timeframe increased from 17% to 27% without an increase in return ED visits. The average time Supertrack patients spent in the ED decreased from 121 to 103 minutes. Personalized reminders demonstrated a significant but short-lived improvement.

**Conclusions:**

We improved the flow of Supertrack patients by decreasing their length of stay and increasing how many were discharged within their anticipated timeframe. Limitations included an unexpected surge in patients with respiratory complaints and staffing and structural constraints preventing the designation of a discrete Supertrack assessment space with dedicated providers. These findings are helpful for institutions seeking to develop an effective fast-track model with limited space and resources.

## INTRODUCTION

Patient crowding in emergency departments (EDs) contributes to delayed time-to-provider, decreased patient satisfaction, increased “left without being seen” rates, and inefficient resource allocation.^1–4^ Nationally, various institutions have implemented structural changes to reduce ED crowding.^5^ Among these include a dual stream or “fast-track” pathway, in which low-acuity patients are identified for rapid evaluation and treatment, typically in a separate, dedicated pre-ED space. Although fast-track structure and criteria vary across institutions, many decrease wait times and lengths of stay (LOS).^6–11^

In 2016, this institution created “Intake”: four rapid assessment rooms where a pediatric emergency medicine (PEM) physician evaluates low-acuity patients presenting through the waiting room and either discharges them or places treatment orders and transfers them to the ED for ongoing care. Of intake patients transferred to the ED, those anticipated to require less than 1 hour of additional care were designated as “Supertrack”—this institution’s version of fast-track—whereas those anticipated to require more than 1 hour of additional care were designated as “Main Bed.” We created these designations to emphasize intake’s role in reducing the time to be seen by a provider. Supertrack was intended to expedite care for patients with more needs than can be accommodated in intake but who shares resources with more acutely ill patients once moved to the ED.

Carney et al^12^ demonstrated that intake decreased the time patients waited to see a provider. However, their report did not include specific findings on Supertrack patients, such as who received a Supertrack designation and how many were discharged within their anticipated timeframe. Currently, many ED providers are unfamiliar with Supertrack and the unique flow of these patients through the ED, which negatively impacts Supertrack’s function as an expedited care pathway.

Before this initiative, 17% of Supertrack patients were discharged within 1 hour of being moved from Intake to the ED. As such, we designed a quality improvement initiative to improve the flow of Supertrack.

## METHODS

This initiative occurred in a tertiary care, level I trauma center pediatric ED that sees approximately 76,000 patients annually. There is no affiliated on-site urgent care department. This institution’s intake space operates from 11 am to 11 pm and sees approximately 17,000 patients annually, 2,000 designated as Supertrack. These hours were determined by the times this institution sees the highest volume of patients in the waiting room.^12^ This study was deemed not human subjects research by the institutional Quality Improvement review panel. There were no conflicts of interest or ethical concerns.

### Project Aim and Core Measures

Our project aim was to improve the flow of Supertrack patients through the ED by increasing the proportion of Supertrack patients being discharged within 1 hour or less from their ED bed assignment from 17% to 50% over 10 months.

Our outcome measure was the percentage of Supertrack patients discharged within 1 hour of receiving their ED bed. This outcome measure was chosen to standardize the duration of ED clinical care for all Supertrack patients, including those who returned to the waiting room or remained in intake for an ED bed to become available.

Process measures included the percentage of Supertrack patients who met Supertrack criteria, average time Supertrack patients spent in the ED, and the percentage of Supertrack patients seen by a second provider in the ED within 30 minutes of receiving an ED bed.

Our balancing measure was the percentage of Supertrack patients who returned for ED care within 72 hours of their initial visit, as an unintended consequence of encouraging prompt discharges might be an incomplete evaluation requiring a return ED visit.

Outcome and process measures were calculated in 2-week intervals. Our balancing measure was calculated monthly. All measures were tracked throughout the intervention period except for the percentage of Supertrack patients seen by a second provider within 30 minutes of receiving an ED bed.

### Interventions

**Fig. 1.**
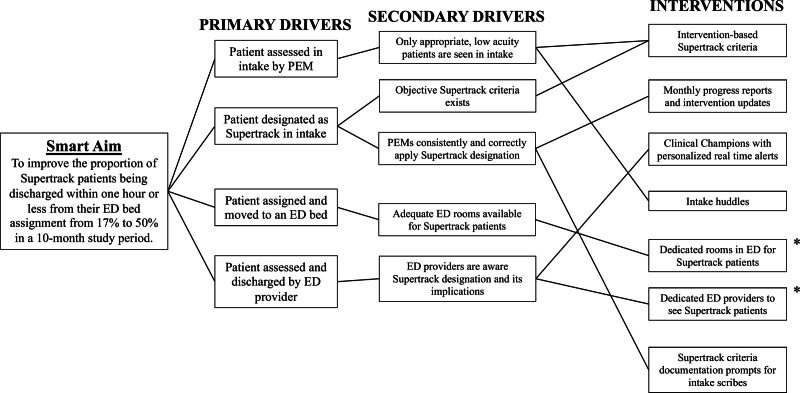
Key driver diagram. *These interventions were not implemented due to external factors.

We developed a process map (**Supplemental Digital Content 1**, http://links.lww.com/PQ9/A597) of Supertrack patients from arrival in the waiting room to discharge from the ED. Next, we convened a multidisciplinary team of key stakeholders. This included PEMs, nurses, advanced practice providers, emergency medicine technicians, scribes, medical trainees, and representatives from the patient-family experience team. This group developed a key driver diagram (Fig. 1) to determine where to focus their efforts and interventions. We implemented interventions in a series of consecutive PDSA cycles, which allowed the team to isolate and study the impact of each intervention.

### Supertrack Criteria—November 1, 2022

We developed new criteria for Supertrack patients based on number and types of interventions required in the ED while maintaining the goal of discharging these patients within 1 hour. New criteria included eight interventions, of which a patient would require up to two to qualify as Supertrack (Table 1). All other patients requiring an ED bed would be designated a main bed patient. Giving antipyretics or collecting respiratory viral testing were not considered interventions, as they did not require significant resources and were ordered too frequently to identify those intake patients with minimal additional needs.

**Table 1.** Supertrack Criteria*

Patient Need	Indication
Albuterol-metered dose inhaler	Mild asthma exacerbation not requiring additional asthma management adjuncts
Antihistamine	Pruritic rashes without signs of anaphylaxis
Ear irrigation	Impacted cerumen precluding an ear exam to diagnose acute otitis media
Enema	Mild constipation
Eye examination with fluorescein	Suspected corneal abrasions in school-age, cooperative children
Urinalysis (obtained via catheter or clean-catch)	Suspected urinary tract infection
x-ray	Musculoskeletal injury without deformity
Zofran and/or oral rehydration therapy	Mild dehydration and oral intolerance from viral gastroenteritis

Patients evaluated in intake who require one or two of the listed interventions for the specified indications qualify as Supertrack patients and should be moved to an ED bed for further management.

* Initial Supertrack criteria included two additional interventions (nasal suctioning and phone subspecialty consultation) that were subsequently removed on April 30, 2023.

Reminders of the new criteria were communicated to all staff via physical signage in the intake space, targeted emails, and presentations to all ED providers. Criteria were amended on April 30, 2023, after an interval chart review revealed two criteria (nasal suctioning and phone subspecialty consultation) consistently resulted in prolonged LOS in the ED.

### Supertrack Updates—November 1, 2022

We presented monthly progress reports that included aggregate performance on all measures, upcoming interventions, and answers to frequently asked questions. These regular updates educated ED providers regarding the purpose of the Supertrack designation and provided accountability for PEMs who work in intake.

### Electronic Health Records Revisions—January 22, 2023

PEM fellows were enrolled as clinical champions and instructed to send targeted reminders to ED providers caring for Supertrack patients via this institution’s electronic health record (EHR) live chat messaging system. These reminders encouraged providers to make a speedy discharge plan for these patients and informed them of the time their Supertrack patient would be in the ED for 1 hour. This messaging system has been integrated into this institution’s EHR for multiple years and is already commonly used for interdepartmental communications.

Additionally, on April 30, 2023, note templates for scribes in intake were amended to include a drop-down menu specifying which Supertrack criteria the patient had.

### Intake Huddles—February 19, 2023

Intake team members gathered at four times throughout the day (11 am, 3 pm, 5 pm, 7 pm) to review waiting room and ED volumes, bed and staffing capacity, and current patient acuity and resource utilization. These huddles provided intake staff with awareness of ED operations that impacted patient flow and helped encourage PEMs to utilize main bed and Supertrack pathways rather than holding patients in intake to complete work-ups and treatments more appropriate for the ED.

### Data Analytics

Data were collected via independent chart review of Supertrack patients and included demographic information, discharge diagnoses, treatment and diagnostic orders, and timestamps of ED bed assignment, second provider assignment, and discharge order. Supertrack patients who were admitted to the hospital, left before completing their visit, and those assigned an ED bed but ultimately discharged from intake were excluded from final analyses. Patients assigned Supertrack while intake was operating outside its routine hours of 11 am–11 pm were also excluded. During these extenuating circumstances, intake beds were used as an expansion of the primary ED space and certain processes defining intake (eg, intake designation and provider staffing) were inconsistent.

We constructed statistical process control charts with QI Macros statistical software (KnowWare International, Inc, Denver, Co.) and followed Nelson rules^13^ to determine special cause variation. We utilized p-charts for all measures except for the average time Supertrack patients spent in the ED, for which we utilized an Xbar S chart.

## RESULTS

**Fig. 2.**
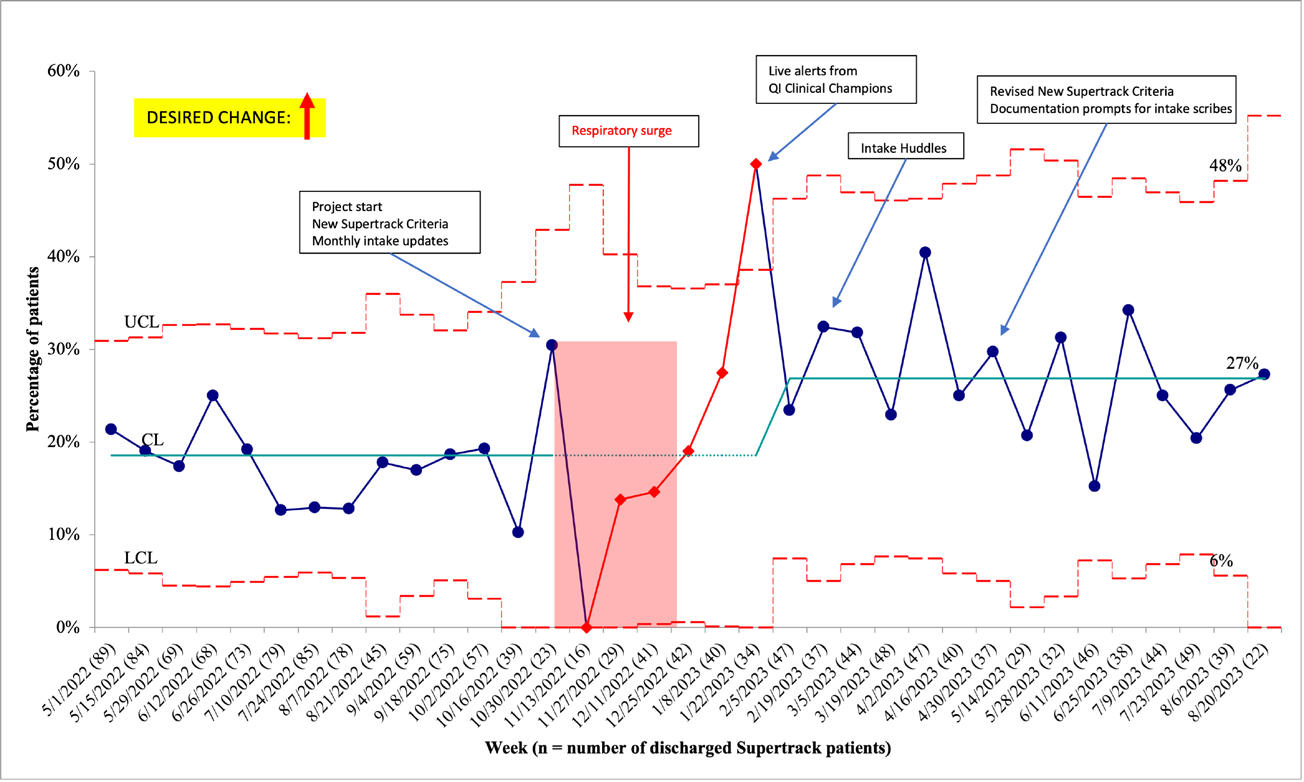
Supertrack patients discharged within 1 hour of ED bed assignment (p-chart).

The average percentage of Supertrack patients discharged within 1 hour of receiving their ED bed increased from a baseline of 17%–27% by the end of this initiative (Fig. 2). We noted special cause variation with an astronomical point above the upper control limit on January 22, 2023. This coincided with the implementation of clinical champions and targeted EHR messaging alerts. Subanalysis of Supertrack patients who met Supertrack criteria (Table 1) demonstrated special cause variation as a run of 10 points above the central line starting on December 25, 2022, increasing from 24% to 33% (**Supplemental Digital Content 2,** http://links.lww.com/PQ9/A598). A similar analysis among Supertrack patients who did not meet Supertrack criteria demonstrated an astronomical point on April 2, 2023, with an increase from 14% to 18% (**Supplemental Digital Content 3,** http://links.lww.com/PQ9/A599). We applied the final iteration of the Supertrack criteria to the months preceding this initiative to determine baseline percentages of Supertrack patients who would have met Supertrack criteria.

**Fig. 3.**
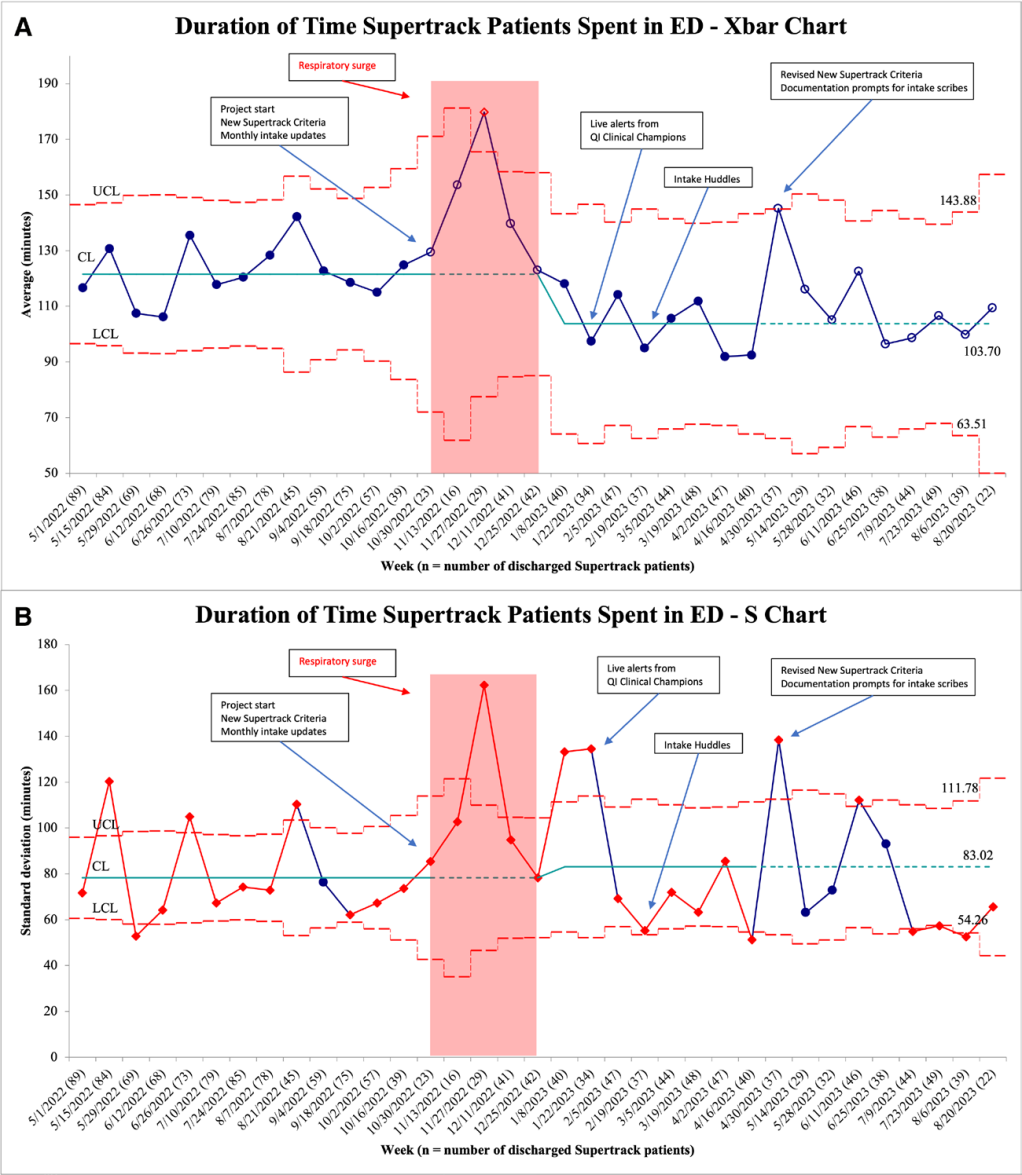
A and B. Duration of time Supertrack patients spent in ED (Xbar S chart).

The average time Supertrack patients spent in the ED was 121 minutes before this initiative (Fig. 3A). Special cause variation occurred on November 27, 2022, with an astronomical point above the upper control limit, which coincided with a sudden surge in patients with respiratory complaints. We felt this was unrelated to ongoing project efforts, so no change was indicated at this point. Special cause variation occurred again from January 8, 2023, to April 16, 2023, with eight consecutive points below the center line. We believed this was in response to project interventions, which triggered a phase change and new center line. The average time Supertrack patients spent in the ED decreased to 103 minutes, a reduction of 18 minutes from baseline (Fig. 3A).

Several points in our Xbar S chart lay beyond our upper and lower control limits (Fig. 3BB). Review of the chart revealed several outliers, all of whom were patients with prolonged observation, imaging studies, or procedures requiring extensive preparation or sedation.

**Fig. 4.**
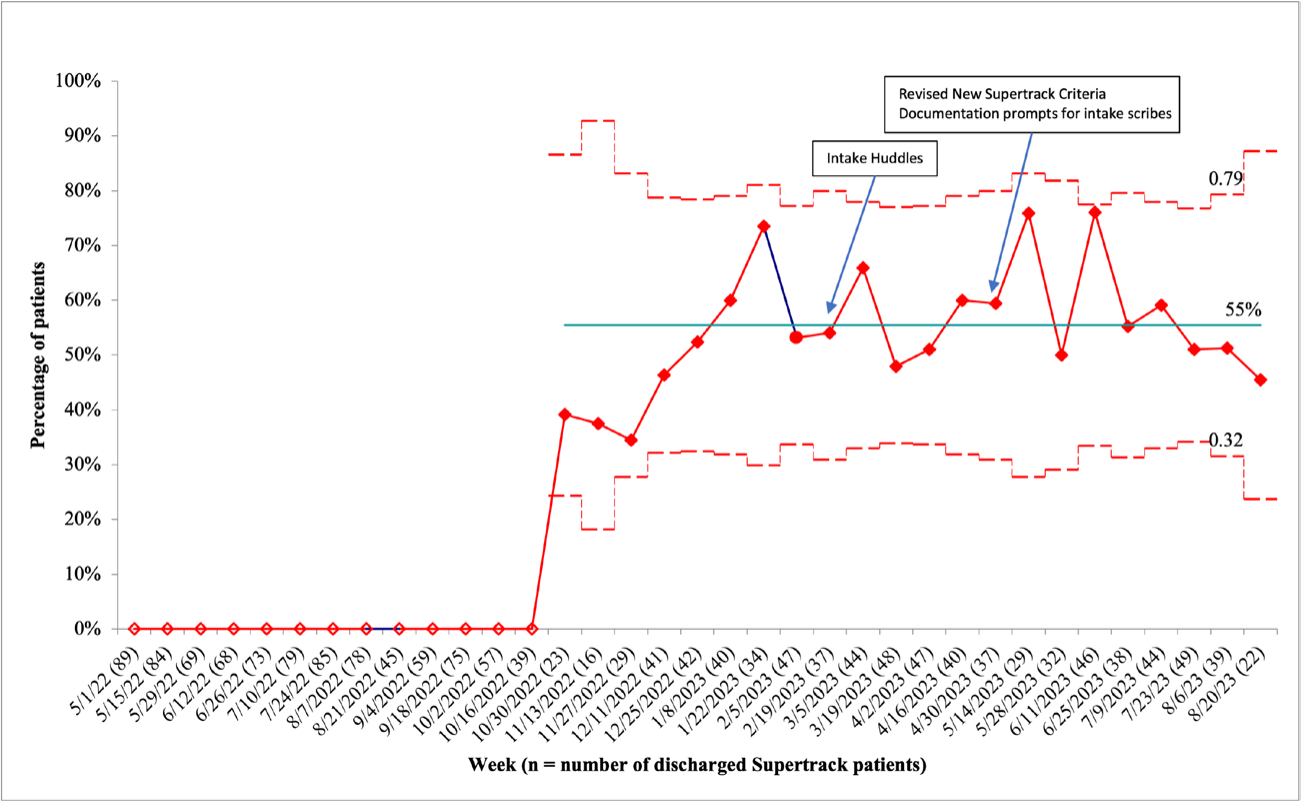
Percentage of Supertrack patients who met Supertrack criteria (p-chart).

Overall, the percentage of Supertrack patients who correctly met Supertrack criteria was 55% (Fig. 4). Data were recorded as absent before November 2022 since, before this, Supertrack criteria was synonymous with the designation itself. The percentage of Supertrack patients with a second ED provider assigned within 30 minutes remained above 95% before and after the start of this initiative.

**Fig. 5.**
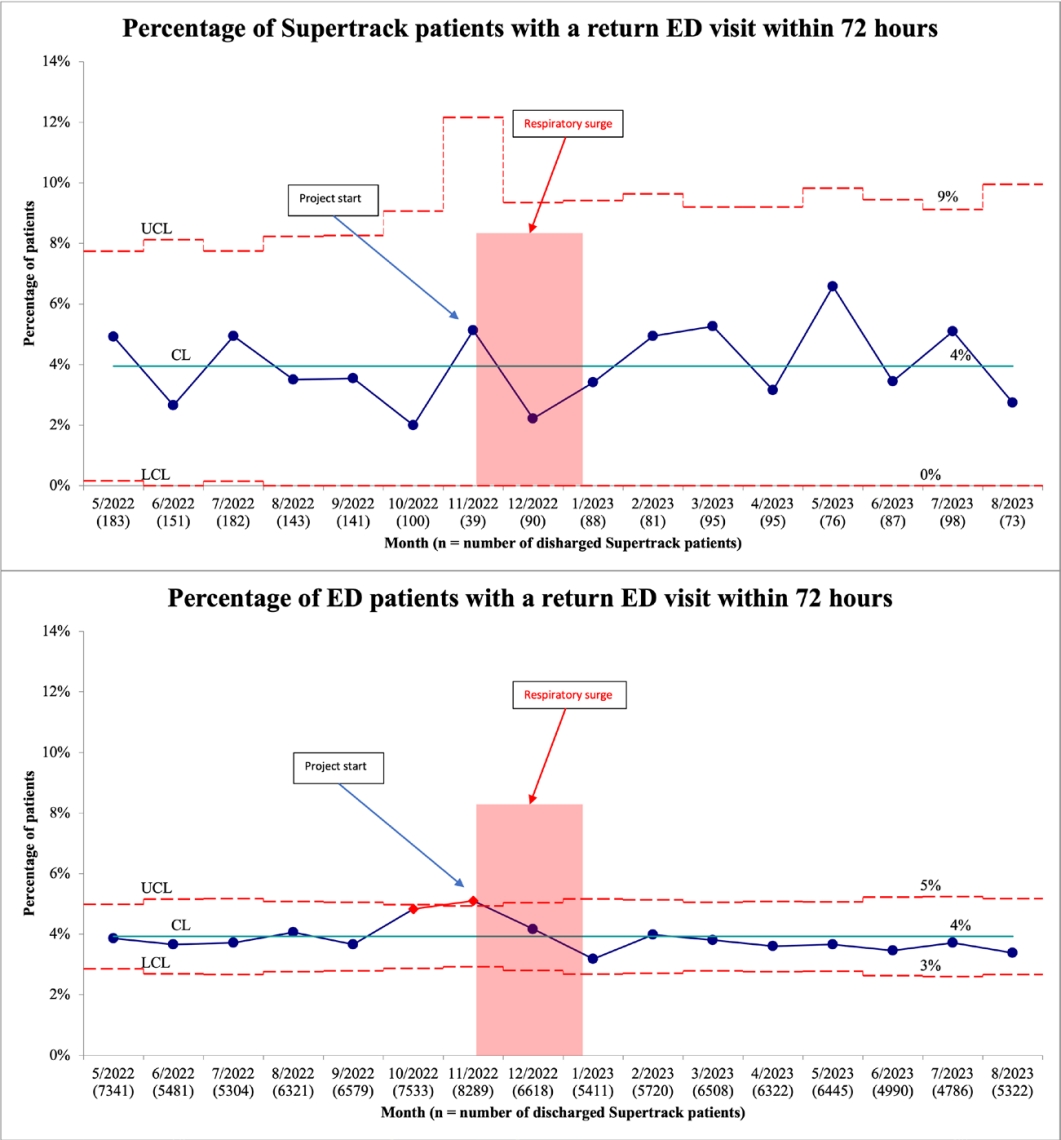
Percentage of Supertrack patients with a return ED visit within 72 hours/percentage of ED patients with a return visit within 72 hours (p-chart).

There was no increase in return ED visits within 72 hours. The number of return ED visits within 72 hours among Supertrack patients was comparable to that of all ED patients (Fig. 5).

Before this initiative, an average of 150 Supertrack patients were discharged from the ED each month. This number decreased after introducing the new Supertrack criteria, with a notable decline during the respiratory surge. The average number of Supertrack patients during the initiative period was 82 per month. Weekly ED volumes during the initiative period varied between 1,000 and 2,000 patients, with higher volumes in November and December 2022 and lower volumes in June and July 2023.

## DISCUSSION

We sought to improve the flow of this institution’s fast-track pathway by increasing the proportion of Supertrack patients discharged within the anticipated 1 hour. Although we did not meet our target aim of 50%, we increased our outcome measure to 27%, observed sustained improvement in our process and outcome measures, and obtained insights that will guide future efforts.

Supertrack is unique in that it attempts to identify patients who require more resources than can be managed in intake alone yet are still anticipated to have a rapid discharge after one or two simple interventions. Prior literature contains many instances of effective fast-track models,^14–19^ though none describe a fast-track integrated within a preexisting front-end system like Supertrack. Supertrack could be considered the “fast-track of fast-track,” as intake itself resembles other fast-track models. Although potentially redundant, given large patient volumes and relatively few resources in intake, Supertrack is necessary to facilitate these patients’ flow by targeting specifically those unable to be immediately discharged.

Before this effort, PEMs incorrectly utilized Supertrack to identify patients with presumed straightforward clinical courses, which did not always result in a rapid discharge. For example, a patient might have all the classic findings of appendicitis and be designated as Supertrack for a confirmatory ultrasound. However, if positive, the patient would require IV antibiotics, a surgical consult, and admission to the hospital for an appendectomy. Ieraci et al^20^ similarly created a fast-track system that selected for apparent clinical need. Although this model demonstrated a decreased LOS, unlike Supertrack, their patients had dedicated resources and staff to help expedite care despite potentially needing time-sensitive work-ups.

Other fast-track models have used objective criteria successfully,^10^ most commonly in the form of frequently encountered, low-acuity diagnoses. Supertrack’s initial criterion of anticipated LOS has not been previously reported, likely due to its subjective nature and how easily LOS is influenced by external factors such as patient volumes, bed availability, and staffing. We introduced novel criteria (Table 1) not seen in prior literature to identify patients who could be discharged quickly from the ED. These criteria did not include demographic factors such as primary language spoken, sex, gender, or payer status to ensure equitable selection. We utilized intervention-based criteria rather than specific diagnoses for multiple reasons. We took this approach because Supertrack patients often did not have a definitive diagnosis at the time of Supertrack designation. Second, although diagnoses range in severity and management course, intervention-based criteria emphasize a speedy clinical course over a specific clinical diagnosis.

An improvement in our outcome measure among Supertrack patients who met the criteria suggests that stricter criteria did not simply select patients who would have been discharged quickly regardless of other clinical factors, but rather, these criteria had a positive impact in facilitating discharges within the expected timeframe. Importantly, adherence to Supertrack criteria was 55%, indicating room to refine this process. Given how long Supertrack had existed before this effort began, long-held personal practices were difficult to change.

Our most successful intervention was using clinical champions to send reminders to ED providers caring for Supertrack patients. The personalized nature of these reminders made them more impactful in changing individual behaviors. Additionally, these alerts raised awareness of how long Supertrack patients had already spent in the ED, information not easily accessible elsewhere in their EHRs. Reminders were well-received by ED providers, as many were unaware that these patients had expectations for timely discharges previously set. Interestingly, Gill et al^21^ found that hand-offs between ED clinicians was one of the strongest predictors of prolonged LOS among fast-track patients, which could explain many of the struggles with Supertrack, as essentially every patient is handed-off to another provider. We believe this intervention may have had a mitigating effect by calling attention to this transition and overtly prompting an expeditious discharge. These reminders had a short-lived effect, likely because of the effort it took to create and send these messages. Poor adherence to Supertrack criteria also may have contributed to this waning effect, as patients with complex needs were uncommonly discharged quickly, regardless of their ED providers’ efforts. However, this success suggests that an automated messaging system in conjunction with improved adherence to Supertrack criteria could be effective.

We believe Supertrack volumes decreased after the onset of this initiative because of stricter criteria as well as unfamiliarity with the Supertrack pathway. These lower volumes suggest that, although our outcome measure improved, the absolute number of Supertrack patients discharged within 1 hour of receiving an ED bed may not have changed significantly. However, the average time that Supertrack patients spent in the ED decreased by 18 minutes throughout this initiative. With an average of 82 Supertrack patients per month, this is equivalent to approximately 24 hours of ED time saved each month. Importantly, these improvements were not accompanied by a significant increase in 72-hour return visits. These findings suggest an overall improvement to the flow of Supertrack patients not captured by our outcome measure.

The onset of this initiative coincided with an unexpected respiratory surge from November to December 2022.^22^ Although our interventions could have been useful at this time, this surge was particularly severe and distinct from other seasonal surges seen by pediatric EDs. During this time, patient volumes far exceeded bed and staffing capacities. An increasing number of hospitalized patients boarding in the ED drastically limited bed availability and cutoff patient flow through the department. Receptiveness to interventions was also decreased, as institutional pressure on ED staff to prioritize triage and throughput made changing practice patterns difficult. This likely dampened positive impacts from interventions, which may have affected outcome and process measures.

This project has several limitations. This was a single-institution QI project with a fast-track model integrated within its unique intake design, making exact replication across other institutions difficult. However, interventions reported here could be easily adapted to fit within other ED models. Additionally, we could not create a dedicated Supertrack physical space or have dedicated Supertrack providers, both of which were anticipated as high-impact interventions by key stakeholders. Staffing shortages limited interventions given competing demands on essential intake nursing and emergency medicine technician staff. Finally, Supertrack data were not easily accessible and required manual extraction, which delayed analyses and intervention implementation.

## CONCLUSIONS

This QI initiative improved flow of Supertrack patients by increasing the proportion of these patients being discharged within their anticipated timeframe while also decreasing the time they spent in the ED. Besides recruiting clinical champions, interventions were sustainable and required few resources to succeed. Changing individual practice patterns and ensuring Supertrack patients correctly met a list of objective criteria were challenges that could use ongoing refinement. These findings suggest that implementing automated reminders to ED providers caring for Supertrack patients and strictly enforcing Supertrack criteria may further facilitate speedy discharges for these patients. Lessons learned from this initiative may be helpful to other institutions looking to implement or improve a fast-track pathway when dedicated fast-track resources are limited or unavailable.

## ACKNOWLEDGMENT

The authors thank IHQSE Quality Improvement Writing Group for assisting in manuscript preparation.

## Supplementary Material
