# Supplementary material for: How Super Is Supertrack? Expediting Care of Fast-track Patients through a Pediatric Emergency Department
Source: Pediatr Qual Saf. 2024 Sep 18;9(5):e770. doi: 10.1097/pq9.0000000000000770 (PMC11410333; doi:10.1097/pq9.0000000000000770)
Supplement: Supplementary file 1 [file pqs-9-e770-s001.pdf]

SDC, Process Map of a Supertrack Patient

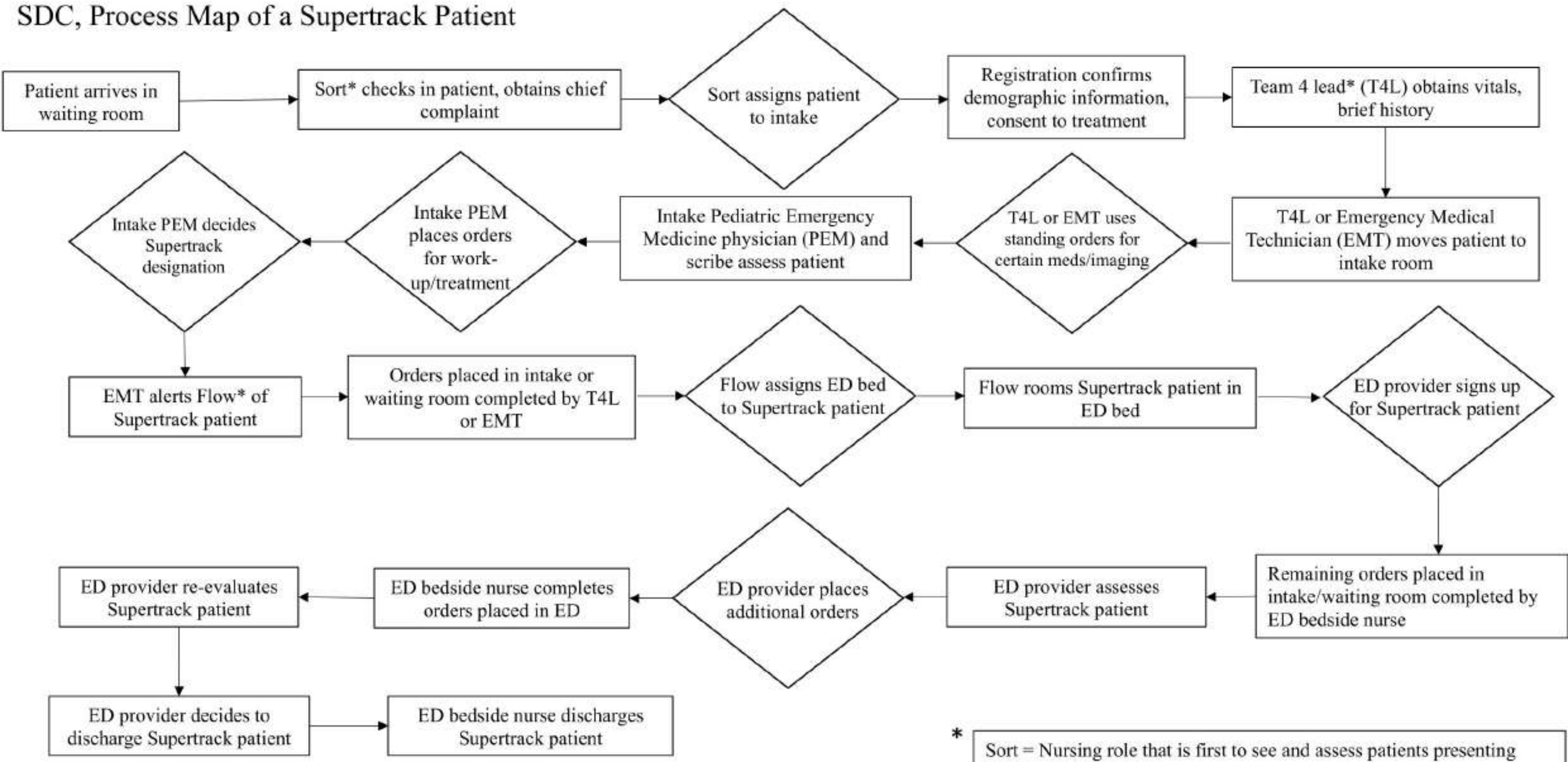

*
Sort = Nursing role that is first to see and assess patients presenting through waiting room
Team 4 Lead = Nursing role that oversees all patients in waiting room
Flow = Nursing or EMT role that manages bed assignments and patient placement

"How Super is Supertrack? A Quality Improvement Initiative to Expedite Care of Fast-Track Patients Presenting through a Pediatric Emergency Department" Lam, D, et al.
