# Supplementary material for: How Super Is Supertrack? Expediting Care of Fast-track Patients through a Pediatric Emergency Department
Source: Pediatr Qual Saf. 2024 Sep 18;9(5):e770. doi: 10.1097/pq9.0000000000000770 (PMC11410333; doi:10.1097/pq9.0000000000000770)

Supertrack patients not meeting Supertrack criteria discharged within one hour of ED bed assignment

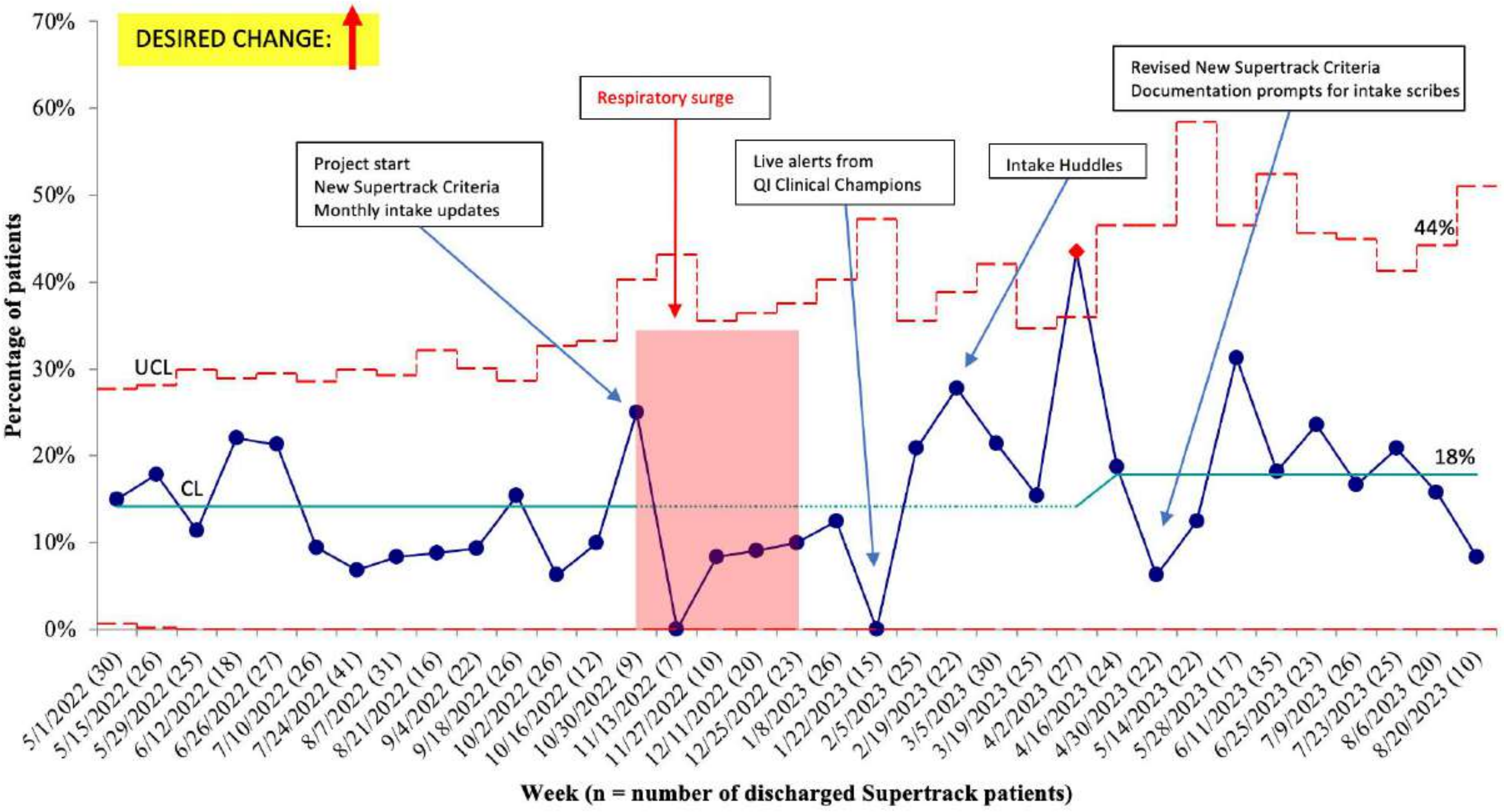

Supplement: Supplementary file 3 [file pqs-9-e770-s003.pdf]
